# Neanderthal and Denisova tooth protein variants in present-day humans

**DOI:** 10.1371/journal.pone.0183802

**Published:** 2017-09-13

**Authors:** Clément Zanolli, Mathilde Hourset, Rémi Esclassan, Catherine Mollereau

**Affiliations:** 1 Laboratoire d’Anthropologie Moléculaire et Imagerie de Synthèse (AMIS), UMR5288 CNRS - Université de Toulouse, Toulouse, France; 2 Faculté de chirurgie dentaire, Université de Toulouse, Toulouse, France; Monash University, AUSTRALIA

## Abstract

Environment parameters, diet and genetic factors interact to shape tooth morphostructure. In the human lineage, archaic and modern hominins show differences in dental traits, including enamel thickness, but variability also exists among living populations. Several polymorphisms, in particular in the non-collagenous extracellular matrix proteins of the tooth hard tissues, like enamelin, are involved in dental structure variation and defects and may be associated with dental disorders or susceptibility to caries. To gain insights into the relationships between tooth protein polymorphisms and dental structural morphology and defects, we searched for non-synonymous polymorphisms in tooth proteins from Neanderthal and Denisova hominins. The objective was to identify archaic-specific missense variants that may explain the dental morphostructural variability between extinct and modern humans, and to explore their putative impact on present-day dental phenotypes. Thirteen non-collagenous extracellular matrix proteins specific to hard dental tissues have been selected, searched in the publicly available sequence databases of Neanderthal and Denisova individuals and compared with modern human genome data. A total of 16 non-synonymous polymorphisms were identified in 6 proteins (ameloblastin, amelotin, cementum protein 1, dentin matrix acidic phosphoprotein 1, enamelin and matrix Gla protein). Most of them are encoded by dentin and enamel genes located on chromosome 4, previously reported to show signs of archaic introgression within Africa. Among the variants shared with modern humans, two are ancestral (common with apes) and one is the derived enamelin major variant, T648I (rs7671281), associated with a thinner enamel and specific to the *Homo* lineage. All the others are specific to Neanderthals and Denisova, and are found at a very low frequency in modern Africans or East and South Asians, suggesting that they may be related to particular dental traits or disease susceptibility in these populations. This modern regional distribution of archaic dental polymorphisms may reflect persistence of archaic variants in some populations and may contribute in part to the geographic dental variations described in modern humans.

## Introduction

Dental morphology shows variability between extant and extinct human groups, but also among modern humans according to a regional distribution [1–3]. Tooth characteristics are shaped by different factors, including of course genetic factors governing dentine and enamel tissue formation [4–8] (http://bite-it.helsinki.fi). But life history parameters, environmental conditions and diets should also be taken into account when studying dental morphostructural evolutionary trends [9, 10]. In the present study, we aimed to determine whether archaic-specific missense variants in tooth proteins from Neanderthal and Denisovan individuals could explain past and present dental morphostructural variability and/or disease susceptibility.

Teeth are composed of three hard tissues (enamel, dentine and cementum) surrounding the pulp and expressing specific non-collagenous proteins including extracellular matrix proteins and proteases involved in the control of mineralization process and crystal deposition during tooth development [11, 12]. Enamel is the hardest external tissue dedicated to mastication. It is formed before tooth eruption by secretory ameloblasts through a two-step process. During the secretion phase of amelogenesis, unique matrix proteins are released (mainly amelogenin, ameloblastin, enamelin, tuftelin and amelotin) and participate to the initiation and growth of hydroxyapatite crystals [11, 13]. During the maturation phase, the matrix proteins are degraded by the specific proteases matrix metalloproteinase 20 and kallikrein 4. Dentin, the “shock absorber” between enamel and pulp, is produced by odontoblast cells lying at the periphery of the pulp where they also play a role in defense and sensory transmission [11]. The dentin organic matrix is essentially composed of collagens but it also contains some proteins controlling dentin mineralization such as the dentin matrix acidic phosphoprotein 1 and the dentin sialophosphoprotein-derived proteins, dentin sialoprotein, dentin glycoprotein and dentin phosphoprotein involved in the nucleation and growth of mineral crystals. The cementum is a thin mineralized layer produced by cementoblasts. It covers the tooth root and maintains its attachment to the alveolar bone. It also acts as a microbial barrier [11, 14]. The composition of the cementum organic extracellular matrix shows similarities with that of the alveolar bone. It is rich in growth factors and glycoproteins that participate in periodontal tissue repair and regeneration after damage. The cementum protein 1 is specific to cementum. It plays a role in the early steps of mineralization and in the local regulation of cell differentiation and proliferation [14]. The bone matrix Gla protein is not specific to cementum but is expressed in the cementum mineralization front where it exerts an inhibitory control on calcification essential for proper mineralization.

The selective pressure and adaptation to specific diets is based on a balance between sharp-cutting efficiency, tooth resistance and protection, and likely played a role in the selection of enamel thickness in primates [15–17]. Fossil and extant hominoids represent an extreme example. To the exception of a few hard-feeding Miocene and Pliocene hominids like *Ouranopithecus* [18] and australopiths [19–21], the genus *Homo* exhibits thicker enamel, with little variation through the course of its evolution during the Pleistocene [20, 22, 23]. However, some human groups like Neanderthals tend to show a thinner enamel [22, 24]. Thinner enamel is also observed for the c. 1 million-years-old Eritrean *H*. *erectus/ergaster* dental sample from Buia and Mulhuli Amo [25].

Many other traits distinguish Neanderthal and modern humans [1, 22, 26, 27]. Neanderthals exhibit a particular anterior dental morphology characterized by a higher degree of robustness and labial convexity, and a higher frequency of shovel-shaped incisors with a marked tuberculum dentale (in the upper incisors), compared to modern humans or African hominin fossils [1, 28]. The Neanderthal post-canine teeth also display a number of typical traits, including a high frequency of accessory features, the lingual displacement of the hypocone, a well-developed mid-trigonid crest (also expressed at the enamel-dentin junction level), centrally set protoconid and entoconid dentin horns, taurodontic roots and enlarged pulp cavity [1, 27, 29]. However, Neanderthals share with modern humans a tendency to reduce their posterior dentition [28] and a similar molar root growth timing [27], even if the tooth crowns tend to show faster dental maturation in Neanderthals [30]. But the frequency of accessory traits and their level of expression, such as for the middle trigonid crest or the centrally shifted dentin horns, are generally much lower in modern humans, at both the outer enamel surface and at the enamel-dentin junction level [27, 29, 31]. Regarding the Denisovan hominins, another extinct human group recently identified by ancient DNA sequencing, dental morphological variability is less documented as only 2 molars have been described, Denisova 4 and 8 [32, 33]. These teeth exhibit distinct primitive morphological characteristics, including the presence of numerous accessory cusps. They are larger than the molars of Neanderthals, modern humans and Asian archaic hominins such as *Homo erectus*, but share with the later a trapezoidal shape [32, 33]. The enamel-dentin junction of the maxillary molar Denisova 8 exhibits a unique occlusal topography, with a large hypocone, a well-developed metaconule and hypercomplex expression of accessory crests and cuspules [33]. However, no information on enamel thickness is available to this date on the Denisovan hominin teeth.

The shape of the outer enamel surface is determined by the enamel-dentin junction through growth of the enamel cap [21, 34]. Polymorphisms in genes or proteins involved in the formation of this tooth region are therefore expected to participate in tooth variability. A number of mutations in the proteins involved in the mineralization (AMEL, AMBN, AMTN, ENAM) or the maturation processes (DSPP, KLK4, MMP20) of enamel and dentin are indeed associated with particular enamel phenotypes or tooth defects, or create susceptibility to dental disorders such as amelogenesis imperfecta (AI), dentinogenesis imperfecta (DI)/dysplasia (DD) or caries [12, 16, 35–42] (Table 1). Recent genetic evidence suggests that enamelin, in tandem with regulation of the processing of the enamel matrix proteins by MMP20, is linked to the evolution of enamel thickness in humans [16]. Conversely, a non-synonymous polymorphism in the enamelin protein, T648I (rs7671281), is associated with a thinner enamel in living humans [15]. In addition, a Neanderthal variant in the post-transcriptional repressor miR-1304 leading to a reduced expression of enamelin and amelotin genes could explain the variation in enamel thickness between Neanderthals and modern humans [26]. Moreover, external tooth morphology may be also affected by a single protein mutation. This is the case of the Asian-specific non-synonymous polymorphism (V370A, rs3827760) in the ectodysplasin A receptor gene (EDAR) which is associated with the expression of shoveling and double-shoveling in upper first incisors, a particular trait of extant Asian populations [43]. This variant, in addition to T648I (rs7671281) in the enamelin protein, emphasizes the role a single amino acid change may play in tooth morphostructure.

**Table 1:** Main non-collagenous extracellular matrix proteins present in tooth hard tissues.

Gene	Protein		Function	Phenotype linked to polymorphism in humans
Name	Name	Uniprot Access.N°		
**AMBN**	**Ameloblastin**	Q9NP70	Structural constituent of enamel, involved in mineralization	AI [40]
**AMELX**	**Amelogenin X**	Q99217	Structural constituent of enamel, involved in mineralization	Hypoplasia, AI [12, 42]
**AMELY**	**Amelogenin Y**	Q99218	Structural constituent of enamel, involved in mineralization	
**AMTN**	**Amelotin**	Q6UX39	Structural constituent of enamel, involved in mineralization	Hypomineralization, AI [41]
**CEMP1**	**Cementum protein 1**	Q6PRD7	Structural constituent of cementum	
**DMP1**	**Dentin matrix acidic phosphoprotein 1**	Q13316	Structural constituent of dentin	
**DSPP**	**Dentin sialophosphoprotein**	Q9NZW4	Structural constituent of dentin, involved in mineralization	DI, DD [12, 38]
**ENAM**	**Enamelin**	Q9NRM1	Structural constituent of enamel, involved in mineralization	Enamel thickness [15] Hypoplasia, AI [36, 37, 42]
**KLK4**	**Kallikrein-4 (Enamel matrix serine proteinase 1)**	Q9Y5K2	Involved in the maturation of enamel matrix proteins	Hypoplasia, AI [12]
**MMP20**	**Matrix metalloproteinase-20 (Enamelysin)**	O60882	Involved in the maturation of tooth matrix proteins	Hypoplasia AI [12]
**MGP**	**Matrix Gla protein**	P08493	Structural constituent of bone and tooth, negative regulator of mineralization	
**ODAM**	**Odontogenic ameloblast-associated protein**	A1E959	Structural constituent of enamel	
**TUFT1**	**Tuftelin**	Q9NNX1	Structural constituent of enamel	

AI: amelogenesis imperfecta, DD: dentinogenesis dysplasia, DI: dentinogenesis imperfecta

In light of these data, the present study aimed to investigate whether tooth protein variants in archaic hominins could provide valuable molecular information to help understand past and present human dental morphostructural variability. To do this, the Neanderthal and Denisovan sequences of the main non-collagenous extracellular matrix proteins of tooth hard tissues (listed in Table 1) were retrieved from publicly available databases [44–46] and were compared with modern human proteins. The detected variants were analyzed for their ancestry, their putative functional impact, and their frequency and regional distribution in living populations, based on informations collected from online databases and the literature. Moreover, finding archaic polymorphisms in present-day humans may enable us to trace back the legacy from archaic admixture with anatomically modern humans (AMH) some 50 000 years ago [47], bringing molecular clues to explain particular dental morphologies or disorders in living populations.

## Methods

The coding sequences of 13 non-collagenous proteins characteristic of tooth tissues (listed in Table 1) were extracted from the publicly available exome database [44] that includes three Neanderthal specimens, the Altaï pedal phalanx (Altaï Mountain Cave, Siberia) and the bone fragments Vi33.15 (Vindija cave, Croatia) and SD1253 (El Sidron cave, Spain). The sequences were aligned to the reference *Homo sapiens* protein sequences in Uniprot (http://www.uniprot.org/). In addition, the tooth protein variants in Denisova [45] and in the incomplete reads of other Neanderthal individuals were searched in the Neandertal Genome Analysis Consortium Tracks on the UCSC Genome Browser website (https://genome-euro.ucsc.edu/; GRCh37/hg19 Assembly) [48]. Among the 13 proteins, amelogenins, particularly the Y-encoded form (AMELY), could not be correctly analyzed because of a poor sequence coverage. Likewise, the sequence of the cementum protein 1 was only available in the Denisova genome. For each variant found, the amino acid change was compared with the chimpanzee (*Pan troglodytes*) and gorilla (*Gorilla gorilla*) proteins in Uniprot in order to assess ancestry. The frequency and the putative phenotype of the protein polymorphism in present-day people was searched in the public Ensembl project database (http://www.ensembl.org/; Ensembl release 84-March 2016) [49]. The geographical distribution maps of the dental protein variants were generated by querying each polymorphism in the online Geography of Genetic Variants Browser (http://popgen.uchicago.edu/ggv/) that compiles Single Nucleotide Variant information from public genomic datasets such as the 1000 Genome Project Consortium [50].

In the present study: the term archaic refers to the extinct hominins Neanderthal and Denisova, the term ancestral indicates that the variant is shared with chimpanzee and gorilla; conversely, the term derived indicates that the variant is not ancestral and differs from apes.

## Results

The alignment to the reference human genome of the protein sequences of the 13 tooth non-collagenous extracellular matrix proteins of the archaic hominins (Neanderthals and Denisova) provided a total of 16 non-synonymous polymorphisms in 6 proteins (Table 2). They are essentially found in Denisova and the Neanderthal individuals Altaï and Vi33.15, owing to their highest sequence coverage. The alignment with the sequences of chimpanzee and gorilla indicates that only two amino acid changes, K55E (rs13331643) in CEMP1 and T127A (rs4236) in MGP are common with apes (Table 2). These variants are also present in modern humans (7% and 39% frequency, respectively) and likely corresponds to ancestral variants. All the other archaic amino acid changes (Table 2) correspond to derived variants found only at low frequencies (< 1%) in living populations, except for the enamelin substitution T648I (rs7671281) which is a major polymorphism today (90%). Conversely, the major alleles in present-day humans are shared with apes, except K55 in CEMP1 that could be considered as modern-derived, and I648 in enamelin, which is common to modern and archaic humans and could be considered as *Homo*-specific. Archaic hominins thus appear to have expressed particular teeth protein variants with likely a high frequency as some of them are shared by several archaic individuals although unrelated in time and geographic location (Table 2). A detailed description of these non-synonymous polymorphisms and their potential functional impact are discussed below.

**Table 2:** Non-synonymous polymorphisms present in tooth proteins of Neanderthal and Denisova hominins.

	Gorilla	Chimp	Modern humans	Neanderthals							Denisova
Variants identified in archaic hominins			(global worldwide frequency)	Altaï (Russia)	Vindija (Croatia)				Mezmaiskaya (Russia)	El Sidron (Spain)	
					Vi33.15	Vi33.16	Vi33.25	Vi33.26	Mez1		
**Ameloblastin (AMBN)**											
G**78S** (rs143795139) derived	G	G	**S (0.0001)**	G	**S**	G	G	G		G	G
M**273V** (rs564905233) derived	M	M	**V (0.001)**	M	M	M					**V**
**Amelotin (AMTN)**											
R**171H** (rs117133443) derived	R	R	**H (0.0001)**	R	R	R	R				**H**
A**200V** (rs376682442) derived	T	A	**V (0.005)**	A	A			A		A	**V**
**Cementum protein 1 (CEMP1)**											
R**15Q** (rs370735504) derived		R	**Q (0.0001)**					R	R		**Q**
K**55E** (rs13331643) ancestral		E	**E (0.07)**								**E**
R**80H** (rs79379654) derived		R	**H (0.001)**				R				**H**
**Dentin matrix acidic phosphoprotein 1 (DMP1)**											
R**173Q** (rs747698893) derived	R	R	**Q (0.0001)**	R	R		R				**Q**
N**483T** (rs574215585) derived	N	N	**T (0.005)**	**T**	**T**			**T**			N
**Enamelin (ENAM)**											
G**389S** (rs74511578) derived	G	G	**S (0.01)**	**S**	**S**		**S**	**S**			G
T**415I** (rs779308123) derived	T	T	**I (0.0001)**	T	T		T	T	**I**	T	T
R**429H** (rs375758699) derived	R	R	**H (0.0005)**	R	R					R	**H**
T**648I** (rs7671281) derived	T	T	**I (0.82)**	**I**	**I**						**I**
T**951M** (rs561402393) derived	T	T	**M (0.0001)**	T	**M**		**M**	**M**		T	T
**Matrix Gla protein (MGP)**											
A**8V** (rs142330429) derived	A	A	**V (0.01)**	**V**	**V**		**V**	**V**		**V**	**V**
T**127A** (rs4236) ancestral		A	**A (0.39)**	**A**	**A**	**A**			**A**	**A**	**A**

Bold characters indicate the aminoacid and position corresponding to the archaic hominin variant. Empty cell indicates no sequence read available at this position. Yellow: common to archaic hominins; Dark green: specific to Neanderthals; Light green: specific to the Vindija cave; Dark blue: specific to Denisova; Light blue: at least present in Denisova.

### Geographical distribution and frequency of the polymorphisms shared by archaic hominins

**Fig 1:**
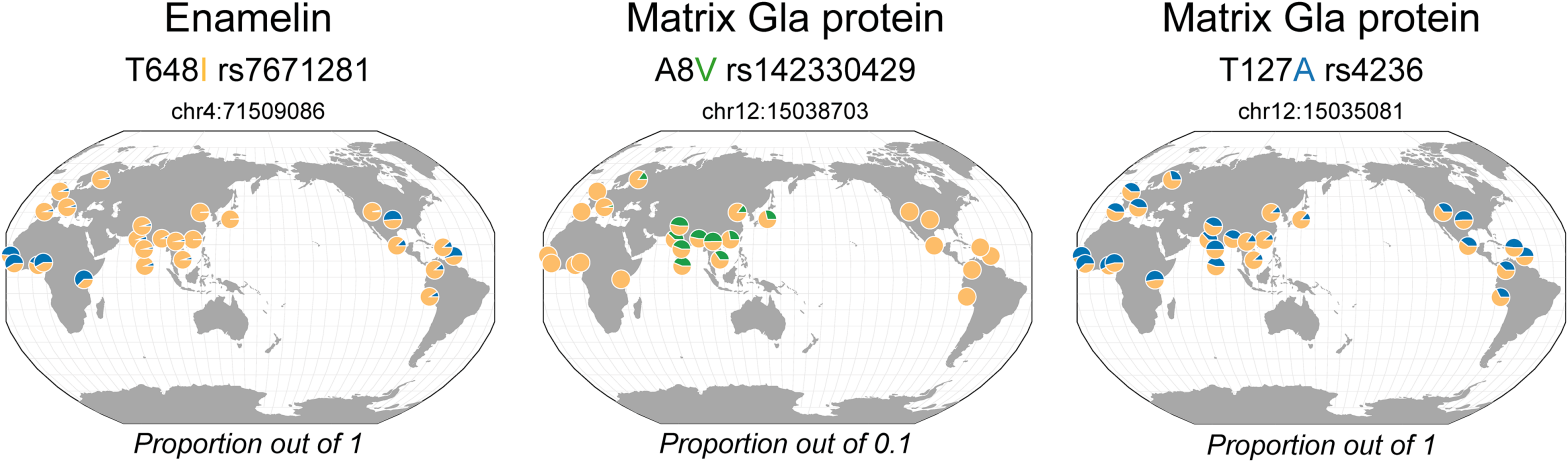
Geographical distribution of the dental protein variants common to archaic hominins in living people. The colored amino acid corresponds to the archaic variant. Yellow corresponds to the major allele; blue and green correspond to minor alleles with a proportion out of 1 and 0.1, respectively. The maps were generated in the Geography of Genetic Variants Browser (http://popgen.uchicago.edu/ggv/) [50].

The 3 non-synonymous polymorphisms common to Neanderthals and Denisova are frequently found in people today (Table 2 and Fig 1). The amino acid substitution T648I (rs7671281) in ENAM corresponds to the major derived variant, nearly fixed out of Africa. T127A (rs4236) in MGP is ancestral and commonly found today, however with the smallest frequency in East Asia. A8V (rs142330429) in MGP is found at a frequency of 3–4% in Asian population only, although a few cases are found in Finland (Fig 1). Interestingly, the two polymorphisms in MGP are detected in Denisovan and Neanderthal individuals arising from nearly all the geographical sites (Russia, Croatia and Spain) indicating a high frequency in archaic hominins. This could be expected for T127A (rs4236) which is an ancestral polymorphism, and suggests that the variant A8V (rs142330429) was probably present in a common ancestor before the separation of Neanderthal and Denisovan lineages that occurred around 381,000–473,000 years ago [51]. Persistence of the A8V polymorphism in East and South Asia may suggest a local segregation in this region.

### Geographical distribution and frequency of the polymorphisms specific to Neanderthal or Denisova

**Fig 2:**
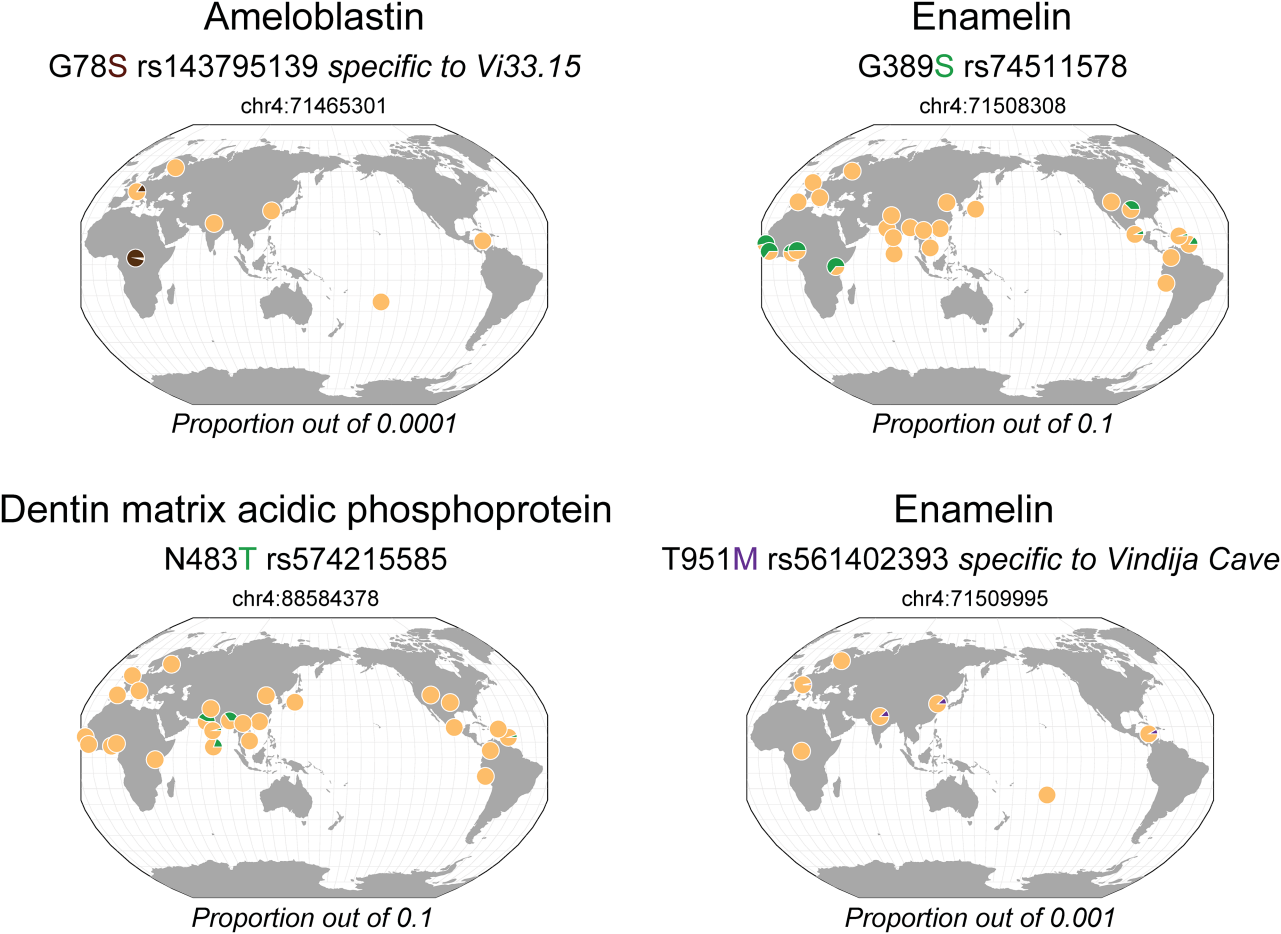
Geographical distribution of the dental protein variants specific to Neanderthals in living people. The colored amino acid corresponds to the archaic variant. Yellow corresponds to the major allele, green, purple and brown correspond to minor alleles with a proportion out of 0.1, 0.001 and 0.0001, respectively. The maps were generated in the Geography of Genetic Variants Browser (http://popgen.uchicago.edu/ggv/) [50].

**Fig 3:**
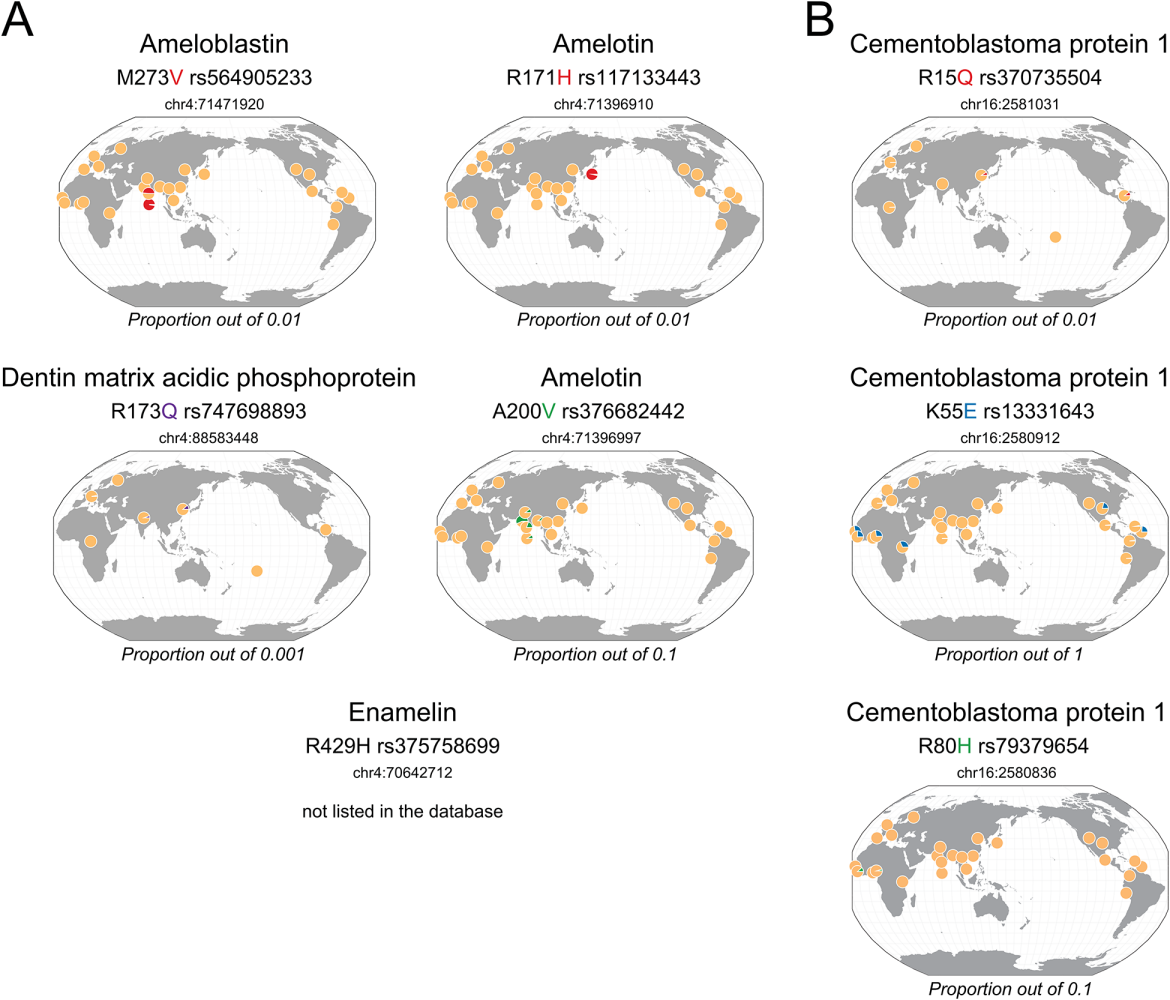
Geographical distribution of dental protein variants A: specific to Denisova, B: at least present in Denisova in living people. The colored amino acid correspond to the archaic variant. Yellow corresponds to the major allele, blue, green, red and purple corresponds to minor alleles with a proportion out of 1, 0.1, 0.01 and 0.001, respectively. The maps were generated in the Geography of Genetic Variants Browser (http://popgen.uchicago.edu/ggv/) [50].

In contrast to the non-synonymous polymorphisms shared by archaic hominins, those specific to either Neanderthals (Fig 2) or Denisova (Fig 3) correspond, for most of them, to non-ancestral variants that are currently present at low or very low frequency in living humans (Table 2). These polymorphisms are, however, mostly found in Africa or in East and South Asia populations (S1 Table).

In Neanderthals, the individuals from the Altaï and Vindija caves express the derived amino acid changes N483T (rs574215585) in DMP1 and G389S (rs74511578) in ENAM. Nowadays, the respective frequency of these variants is 2% in South Asia and 5% in Africa and America (Fig 2). The variant G78S (rs143795139) in AMBN is specific to the Vi33.15 individual and is observed today at very low (≤ 0.01%) frequency in Africa and South Europe. In addition, another substitution in ENAM, T951M (rs561402393), is specific to the individuals from the Vindija cave and corresponds to a rare variant (< 0.1%) present today in South and East Asia as well as South America.

The tooth proteins of the Denisova individual contain a large number of specific polymorphisms: M273V (rs564905233) in AMBN; R171H (rs117133443) and A200V (rs376682442) in AMTN; R173Q (rs747698893) in DMP1 that are essentially found today in the Asian population at a low (1–5%) or very low (0.01%) frequency. In addition, three variants in CEMP1, R15Q (rs370735504), K55E (rs13331643) and R80H (rs79379654), are also detected in Denisova, but cannot definitively be attributed to this lineage in the absence of protein coverage in the Neanderthal individuals (Fig 3B). K55E is a common variant in Africa (26% frequency, Fig 3 and S1 Table).

### Archaic tooth protein variants: Description and discussion

#### Enamelin

It is interesting to note that the majority of the non-synonymous polymorphisms identified in archaic hominins are found in enamelin (ENAM), the protein playing an essential role in the proper formation of enamel and involved in enamel thickness [15]. It has been proposed that the ENAM gene was under positive selection related to diet change during the primate lineage evolution, and some enamelin variants were shown to influence enamel thickness [15–17]. In particular, the derived ENAM amino acid substitution T648I (rs7671281), which is common in sub-Saharan Africa and nearly fixed out-of Africa (Table 2 and Fig 1), is associated with a thinner enamel [15] and may explain the difference in dental size between populations with African and non-African origins [2, 3, 52]. Interestingly, this polymorphism is present in both Neanderthal and Denisova hominins and not apes, suggesting that it was carried by a more ancient common ancestor, consistent with positive selection in the *Homo* lineage [17]. This variant may have contributed to the continuous decrease in enamel thickness from Plio-Pleistocene hominins (including *Australopithecus* and *Paranthropus*) to *Homo* [53].

However, the T648I polymorphism by itself cannot fully explain the relatively thinner enamel of Neanderthals as this variant is shared with modern humans that exhibit a thicker enamel [22, 24] and with Denisovans that harbor tooth characteristics more reminiscent to early *Homo* [32, 33]. It is therefore possible that the other polymorphisms specific to each hominin lineage may explain some of these differences. In particular, most of the Neanderthal individuals show accumulation of specific amino acid changes in ENAM (G389S, T951M) that could have contributed, in addition to another Neanderthal specific substitution in DMP1 (N483T, Table 2) and to the miR-1304 polymorphism described by [26], to the singularity of Neanderthal teeth characterized by an apparent thin enamel and particular dentin/enamel proportions [24, 29]. A difference in tooth size has been proposed to explain variation in enamel thickness. Modern humans have smaller molars than Neanderthals but the coronal dentine is disproportionately reduced compared to the enamel cap, resulting in relatively higher enamel thickness values [22, 24]. How proteins may affect the relative proportions of dentin and enamel in relation to crown size is yet unknown. Investigating the influence of the ENAM and DMP1 polymorphisms identified here on dental structure, especially in the Asian population where they are more frequent, could be a mean to get more insights.

Furthermore, the T648I variant in ENAM has been proposed to offer protection against caries since the reverse variant I648T, corresponding to the ancestral and today minor allele, has been associated, in combination with another ENAM polymorphism R736Q, with increased risk of carries in a French children cohort [35]. The advantageous T648I substitution nearly fixed today may have been inherited from the archaic hominins, that show rare sign of caries [54, 55] and low level of defective enamel formation [37]. In addition to susceptibility to caries, mutations in the ENAM gene have been described to cause enamel defects such as hypoplasia (thinner and more irregular enamel) and hypomineralization linked to *Amelogenesis imperfecta* (AI) disorders [37]. Local hypoplastic enamel defect occurs from <1% to >56% of living humans depending on populations, and was rare in Neanderthals [37] and earlier human groups [56, 57].

The 5 non-synonymous ENAM polymorphisms found in archaic hominins (Table 2) are all located in the more variable region encoded by exon 10 of the protein and do not correspond to any described SNP in AI disease [58]. Four of these mutations lead to the addition or the removal of a putative phosphorylation site in the protein. It is interesting to note that one reported case of AI corresponds to the suppression of a phosphorylation site on the S216 residue of ENAM [59], suggesting that the variants identified in the archaic hominins may have a functional impact.

#### Dentin matrix acidic phosphoprotein 1

DMP1 is expressed in odontoblasts and osteoblasts and regulates cell differentiation and biomineralization of dentin and bones. The protein acts as a transcriptional factor regulating the expression of bone and dental genes, and also participates in phosphate homeostasis [11, 60]. Deletion or missense mutation of the protein lead to cartilage malformation, defect in the predentin to dentin maturation, hypomineralization and hypophosphatemia [60]. The Neanderthal (N483T) or Denisova (R173Q) variants have not been shown involved in a particular phenotype or disease. They are located in the C-terminal of DMP1 that is enzymatically-processed to a 57 kDa fragment promoting hydroxyapatite nucleation and containing nuclear localization signals. Interestingly, N483T is located in a highly phosphorylated domain of the protein [60]. DMP1 phosphorylation is important for the control of dentin and bone matrix mineralization. Therefore, the threonine residue introduced by the mutation could potentially create an additional phosphorylation site susceptible to have functional consequence, such as a higher collagen matrix mineralization.

#### Ameloblastin

After amelogenin, ameloblastin (AMBN) is the second most abundant enamel matrix protein. The protein interacts with amelogenin and participates in enamel formation by a not yet elucidated mechanism [11, 13, 40]. Nevertheless, AMBN plays an essential role in amelogenesis as demonstrated by the severe hypoplasia of knock-out mice [61]. Moreover, a deletion in the AMBN gene has recently been involved in a case of AI in a Costa Rica family [40]. Apart from this deletion, the functional consequence of AMBN polymorphisms are poorly documented. The two variants identified in Neanderthal Vi3315 (G78S) and in Denisova (M273V) individuals are not located into any functional or conserved domains of the protein [62].

#### Amelotin

This protein has been recently identified as another, less abundant enamel matrix protein expressed during the maturation stage of amelogenesis and was proposed to promote hydroxyapatite precipitation [13, 41]. As for AMBN, amelotin knock-out mice show enamel defects and deletion of exons 3–6 in the AMTN gene has been associated with AI in a Costa Rica family [41]. Two AMTN amino acid changes (R171H, A200V) were detected in the Denisova individual only and may form a haplotype characteristic of this lineage. The variants are not located in protein domains conserved during mammalian evolution or harboring biological activity [63].

#### Cementum protein 1

CEMP1, recently identified as an extracellular matrix protein, is considered as a marker of cementum [14]. It plays a role in the differentiation of cementoblasts, and in the mineralization process by promoting the deposition and growth of hydroxyapaptite crystals. It may have biological importance for tissue regeneration in periodontal disease. To our knowledge, no CEMP1 polymorphisms, including the variants R15Q, K55E and R80H detected in Denisova (Table 2), have been described associated with a particular phenotype. It can be noticed that the Denisova individual carries 3 substitutions (R15Q, K55E, R80H) that could be considered as a haplotype. K55E and R80H are located in the domain of the protein showing similarity with collagen alpha1 (I) [14] and all 3 mutations cause a change in the charge of the residue that could impact functional interactions. Noteworthy, K55E corresponds to the ancestral variant, whereas the major substitution K55 in living humans is derived and nearly fixed out of Africa (Fig 3B), and likely represents a polymorphism specific to the modern lineage.

#### Matrix Gla protein

MGP is an essential extracellular matrix protein that exerts an inhibitory control on tissue calcification [14, 64]. Functional deficit or reduced expression of the protein lead to tissue over-mineralization. As MGP is expressed in kidney, lung and heart, in addition to bone and teeth, protein disfunction is associated with severe cardiovascular diseases. The nowadays common MGP variant T127A (rs4236) found widespread in the sampled archaic hominins (Table 2) was not associated with subgingival dental calculus [65] but was shown to decrease by 1.4–1.8 fold the risk of kidney stones in Han Chinese or Japanese patients [64, 66]. The T127A substitution suppresses a potential phosphorylation site at the C-terminal end of the protein that could be important for protein activity or for its interaction with other proteins or substrates. The other archaic and derived polymorphism A8V (rs142330429), nowadays restricted at low frequency in Asia and Finland, is not specifically found related to any relevant phenotype, although MGP is found among the 22 proteins inferred to be introgressed from archaic hominins in living humans, and which were sorted in relation to hypoplasia and skeleton abnormalities by phenotype-ontology enrichment tests [44].

## Discussion

The analysis of tooth proteins in archaic hominins reveals 16 non-synonymous polymorphisms in 6 non-collagenous extracellular matrix proteins. Most of them correspond to derived variants for which the ancestral amino acid (common with apes) is in contrast present at high frequency in living people. This is in agreement with the global exome analysis of Castellano et al. [44] showing a higher proportion of derived amino acid changes in archaic compared to living humans and a greater susceptibility to altered protein structure and function. Considering all the Neanderthal missense derived variants, their frequencies are below 10% in living humans [67], which compares with the frequencies observed here for the archaic tooth variants (Table 2). A number of 274 missense polymorphisms for which the ancestral allele is in contrast fixed in modern humans is reported by Prufer et al. [46], and a minimum of 82 derived substitutions in 78 proteins can be estimated from Castellano et al. who sorted the variants by phenotype-ontology enrichment [44]. They found in particular, 10 Neanderthal-specific variants in 6 proteins involved in hyperlordosis, and 26 archaic-specific variants in 22 proteins associated in part with hypoplasia and skeleton abnormalities. Noteworthy, among them the MGP variant A8V certainly deserves to be considered in future genome wide association studies including dentition, and in the context of particular regional distribution (see below).

**Fig 4:**
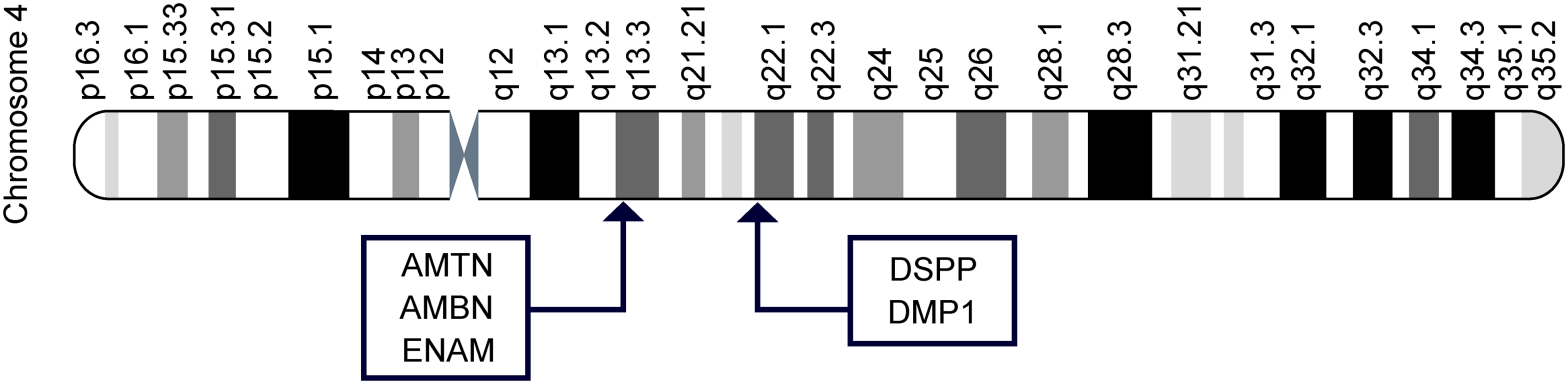
Localization of tooth protein genes in two clusters on chromosome 4.

Most of the amino acid changes are found in the dentin and enamel proteins that are encoded by genes located in two close clusters on the chromosome 4 (Fig 4) and supposed to derive from an ancestral gene encoding a secretory calcium-binding protein [68]. These clusters fall apart from known haplotypes on chromosome 4 and do not correspond to candidate Neanderthal or Denisovan gene flow regions in non-African living humans [69, 70]. However, a signal of archaic introgression in the locus 4qMB179 on chromosome 4 has been evidenced in Central African populations, and is supposed to result from admixture with an archaic *Homo* some 35 kya [71]. In this study, the authors analyzed a non-coding intergenic region (>31 kb) within the locus. It would be therefore relevant to test if the region of the chromosome 4 that harbors the dental protein polymorphisms of Neanderthal and Denisova hominins may also reflect the signature of an archaic introgression in Africa. This is even more pertinent since there is evidence, as mentioned above, of positive selection of the ENAM gene [15–17] and since some other non-archaic missense variants in AMBN, ENAM and DMP1 are specifically expressed in Africa (S1 Fig).

Among the 16 archaic polymorphisms identified, only 2 are ancestral K55E (rs13331643) in CEMP1 and T127A (rs4236) in MGP. The CEMP1 variant K55E (rs13331643) is nowadays mostly restricted to Africa with a frequency around 25%, while in the rest of the world the derived modern-specific substitution K55 is the major allele. This suggests that the ancestral variant may have not been favorable to AMH outside Africa or, conversely, that the derived allele was specific to AMH and evolved out of Africa due to a founder effect. The ancestral archaic variant could also explain in part the tendency of people with African origins to have larger crowns and root volume and surface [2, 3, 52, 72]. Indeed, this variant is also found in Neanderthals teeth, characterized by high crown dimensions and frequent taurodontism [73]. The presence of this variant in Denisova is also compatible with such traits. Even if only two molars are reported for this taxon, they have extremely large crowns and Denisova 4 (the only one with fully preserved roots) displays massive diverging roots [33]. The MGP ancestral variant T127A (rs4236) is common (39% frequency) but less frequent in East Asia (around 10% frequency) suggesting that this ancestral allele was not adapted to the regional context, or was more rapidly diluted by admixture with local non-carrier population.

Other than the common worldwide derived ENAM polymorphism T648I (rs7671281) involved in a thinner enamel that is nearly fixed out of Africa and could be considered as *Homo*-specific, all derived polymorphisms are minor or rare alleles found restricted to either Africa or East/South Asia. Their frequencies fall in the range (< 0.1%) of the majority of the other non-archaic tooth protein missense variants (S1 Table). Derived variants with the highest frequencies do not exhibit specific regional distribution (S1 Fig) but, as for low and very low archaic variants, some polymorphisms in AMBN, ENAM, DMP1 and MGP, are distributed in either Africa or Asia (S1 Fig). Compared to Neanderthal missense variants located in proteins inferred to be introgressed [44, 67, 74], the frequencies of archaic tooth polymorphisms are lower (S2 Fig), indicating that the archaic derived variants in tooth proteins were probably not under positive selection, but may reflect a signature of population bottleneck in some regions and could trace persistence of archaic origin in specific populations.

The finding in living Africans of rare derived polymorphisms that were present in Neanderthals (Fig 2: ENAM G389S, 5%) or Denisova (Fig 3: CEMP1 R80H, 0.5%) is difficult to reconcile with the fact that interbreeding of AMH with Neanderthals occurred out of Africa [75, 76]. However, as mentioned above, this could be explained by the inside Africa admixture between the ancestors of AMH and archaic hominins, or by the possible back migration of Neanderthal gene flux [71, 75]. Further genome analysis of the Neanderthal and Denisova polymorphisms in AMBN, ENAM, and CEMP1 certainly deserves attention as they could trace back an African ancestor common to Neanderthals, Denisova and early anatomically modern humans.

The other archaic derived variants (M273V in AMBN, R171H and A200V in AMTN, R15Q in CEMP1, N483T and R173Q in DMP1, A8V in MGP) are mainly found expressed at frequency between 0.1% to 4% in East and South Asia today, in accordance with highest proportion of Neanderthal and Denisova genomes remaining in their genomes and in the genome of South Pacific people [47, 70, 77]. Those variants could be responsible for tooth characteristics particular to Asian living people. Although there seems to be little variation in average and relative enamel thickness in present-day humans [78], Asians do show more complex dental traits than other populations. The main characteristic is a high prevalence of shoveling and double shoveling of the upper incisors that is less frequent in Europeans and Africans, additional cusp 6 and extra distal roots [52, 79]. According to [1], the highest frequencies are found in North Asia and the Americas. The lowest are in Western Eurasia and Sahul-Pacific. For the double shoveling, the highest frequencies are present among Native Americans (55–70%) and North Asia (62.5%) [1, 3]. The lowest are found in Southern Africa (0%) and sub-Saharan Africans (1.3%). Shovel-shaped incisors and crown size have been associated with an Asian-specific non-synonymous polymorphism in the Ectodysplasin receptor gene (EDAR) involved in hair thickness but absent in European, African and Denisovan populations [43, 79]. Interestingly, the shoveling phenotype is observed in *Homo erectus* and Neanderthal teeth [28, 79]. The EDAR variant does not explain all the Asian dental traits, and other genetic factors are likely involved, in particular regarding the development of the hypoconulid [43]. In support, the post-transcriptional repressor miR-1304 ancestral allele present in Neanderthals and today restricted to Asia (6% frequency in South Asia), which is responsible for the lower expression of the dental proteins enamelin and amelotin, may also shape the dentition or contribute to a higher risk of amelogenesis imperfecta in Asian populations [26]. In this regard, the polymorphisms common to modern East/South Asians and to Neanderthal or Denisova individuals, identified here in AMTN, DMP1 and MGP, could also have an influence on tooth structure or dental health in these populations. Investigating the functional consequences of these variants in relation to the prevalence of dental and other diseases in the Asian population certainly deserves interest, in particular for A8V in the protein MGP that is found associated in phenotype-ontology test with skeleton abnormalities, cardiovascular and metabolism diseases, hair and morphological traits [44].

In conclusion, this study presents a list of archaic tooth proteins variants with potential matter of investigation for those who are interested in the relationships between protein polymorphisms and dental morphostructure, taxonomic determination of archaic lineages, or characterization of particular traits or disease susceptibility in diverse present-day populations [72]. However, further biochemical and functional exploration or genome wide association studies of the polymorphisms are now required. The present study also points to the specific regional distribution of rare dental polymorphisms with archaic origin around the world, bringing some support to the geographic morphological patterns described earlier [1, 3]. Deciphering the functional impact of these polymorphisms on teeth could be relevant for dental practitioners to adapt their treatment in view of personalized medicine. It also illustrates that our knowledge of the functional consequence of the human polymorphism diversity is essentially based on population with European ancestry, and that informative data from other ethnic groups should also be included [80].

## Supporting information

S1 Table Tooth protein missense variants with a frequency > 0.001 (1000 Genome or ExAC, http://www.ensembl.org). Archaic variants are indicated in bold characters. AFR: Africa AMR: America EUR: Europe EAS: East Asia SAS: South Asia.
(PDF)

S1 Fig Geographical distribution of non-synonymous variants (frequency > 0.001) in tooth proteins. For clarity in the ENAM diagram, the major variant T648I was not considered. * Archaic variants.
(PDF)

S2 Fig Frequency of archaic missense variants in tooth proteins compared to proteins infered to be introgressed. ^1^CUL7, HSPG2, NEB, FRAS1, FREM2 are found in Neanderthal or archaic (Neanderthal + Denisova) introgressed regions (Castellano et al. 2014). ^2^ZNF365 and SLC16A1 are found associated with Crohn's disease in Ashkenazi Jews and Type II diabetes in Latinos, respectively (Sankararaman et al., 2014). ^3^SIPA1L2 is present in haplotype with signature of adaptive introgression in Asia (Vernot and Akey, 2014). ^#^ Ancestral variants.
(PDF)

## Abbreviations

AI: amelogenesis imperfecta
AMBN: ameloblastin
AMELX: chromosome X encoded amelogenin
AMELY: chromosome Y encoded amelogenin
AMH: anatomically modern humans
AMTN: amelotin
CEMP1: cementoblastoma-derived protein/cementum protein 1
DMP1: dentin matrix acidic phosphoprotein 1
DSPP: dentin sialophosphoprotein
EDA: ectodysplasin-A
ENAM: enamelin
KLK4: kallikrein-4/enamel matrix serine proteinase 1
MGP: matrix Gla protein
MMP20: matrix metalloproteinase-20/enamel metalloproteinase
TUFT1: tuftelin
