# Supplementary material for: Neanderthal and Denisova tooth protein variants in present-day humans
Source: PLoS One. 2017 Sep 13;12(9):e0183802. doi: 10.1371/journal.pone.0183802 (PMC5597096; doi:10.1371/journal.pone.0183802)
Supplement: S1 Table — Archaic variants are indicated in bold characters. AFR: Africa AMR: America EUR: Europe EAS: East Asia SAS: South Asia. (PDF) [file pone.0183802.s001.pdf]

**AMBN** (over 209 missenses)	Allele frequency of the variant					
	**All**	**AFR**	**AMR**	**EUR**	**EAS**	**SAS**
S41N (rs546778141) derived	0.16	0.07	0.19	0.24	0.3	0.01
M76V (rs200620822) derived					0.001	
M76I (rs145190129) derived	0.002	0.01				
G78V (rs145763811) derived	0.005	0.016	0.006			
**G78S (rs143795139) derived**		0.0001				
S102Y (rs145745749) derived	0.006	0.02	0.006			
P125H (rs180819235) derived			0.001			
E142K (rs550317645) derived						0.001
V181A (rs189846864) derived	0.001		0.004			
D182Y (rs543637559) derived				0.001		
R222H (rs142755020) derived					0.001	
P230S (rs369135444) derived	0.001					0.003
V244M (rs201976575) derived	0.01					0.05
A255V (rs7439186) derived	0.12	0.15	0.073	0.065	0.15	0.14
G268R (rs146167261) derived	0.002			0.002		0.006
**M273V (rs564905233) derived**	0.001					0.003
G276R (rs201299436) derived					0.001	
M285I (rs115723025) derived	0.034	0.12	0.01			
G288S (rs568675718) derived	0.001					0.004
P293S (rs200796492) derived			0.001			
H294Q (rs113506649) derived	0.02	0.065	0.01			
G300S (rs139319140) derived	0.002	0.007			0.001	
E317A (rs150017698) derived	0.003	0.01	0.001			
M328T (rs538513467) derived			0.001			
P329L (rs140331879) derived	0.004		0.003		0.02	0.001
A351T (rs141401324) derived		0.001				
L354P (rs72654387) derived	0.02		0.024	0.06		0.01
G363S (rs543838539) derived					0.001	
V378I (rs76503327) derived	0.024	0.09	0.006			
L391S (rs141535122) derived				0.002		
T416A (rs527740107) derived		0.001				
T417M (rs146171297) derived						0.001
E436K (rs149937358) derived	0.001			0.002	0.003	0.001
A441T (rs144829376) derived	0.001	0.002		0.001		

**AMTN** (88 missenses)	Allele frequency of the variant					
	**All**	**AFR**	**AMR**	**EUR**	**EAS**	**SAS**
R15W (rs35286445) derived	0.11	0.007	0.11	0.16	0.19	0.14
P18S (rs35426053) derived	0.006	0.02	0.001			
P33L (rs199658784) derived						0.001
N45S (rs7660807) derived	0.1	0.006	0.06	0.1	0.2	0.17
V47F (rs192563032) derived	0.001	0.007				
S50P (rs34803339) derived	0.03	0.07	0.01		0.001	0.04
G78S (rs151041998) derived	0.001			0.004		0.001
G88E (rs146006787) derived	0.001		0.003	0.004		
L89M (rs560519245) derived						0.001
A109T (rs201205218) derived	0.001		0.001	0.001		0.003
H129Y (rs148416277) derived		0.002				
G166E (rs565052404) derived		0.001				
R171C (rs201349837) derived	0.001	0.002			0.001	
**R171H (rs117133443) derived**						
G177V (rs61755875) derived	0.02	0.06	0.004	0.01	0.005	
D181N (rs185210496) derived					0.001	
**A200V (rs376682442) derived**	0.004					0.02

**CEMP1** (over 143 missenses)	Allele frequency of the variant					
	**All**	**AFR**	**AMR**	**EUR**	**EAS**	**SAS**
A11G (rs547680075) derived						0.001
**R15Q (rs370735504) derived**			0.001		0.001	
S19F (rs201463378) derived			0.001			
T27I (rs563820819) derived		0.001				

C28R (rs200449794) derived					0.001	
G50V (rs574778073) derived					0.001	
**K55E (rs13331643) ancestral**	0.07	0.26	0.02	0.002		0.001
**R80H (rs79379654) derived**	0.001	0.005				
R81H (rs200977329) derived			0.003			
A103S (rs535834483) derived	0.002					0.008
A107G (rs76722821) derived	0.008		0.02	0.03		0.002
R113K (rs202046694) derived			0.001			
S120P (rs200124887) derived		0.001				
P164L (rs531558523) derived						0.001
V165I (rs563584391) derived						0.001
P173Q (rs543906255) derived		0.001				
V182L (rs189095388) derived				0.002		
P214L (rs185302486) derived					0.001	
R216Q (rs141294706) derived		0.002				
T223M (rs558847727) derived						0.001
R225C (rs181209921) ancestral					0.001	
R225H (rs576590180) derived			0.001			
G234S (rs536394645) derived			0.001			
T238K (rs61746888) derived		0.001		0.001		
**DMP1** (over 216 missenses)	**Allele frequency of the variant**					
	**All**	**AFR**	**AMR**	**EUR**	**EAS**	**SAS**
R21K (rs552859119) derived				0.001		
S27P (rs150105108) derived					0.001	
T42P (rs369550864) derived	0.001				0.004	
E49G (rs200704471) derived		0.001				
E60D (rs546062219) derived						0.001
S69C (rs10019009) derived	0.29	0.26	0.33	0.23	0.43	0.21
D80N (rs567911405) derived			0.001			
A88V (rs144773084) derived		0.001				
F91L (rs556512826) derived						0.001
T113I (rs145164698) derived		0.002				
D141N (rs144580319) derived	0.001	0.002				
D142Y (rs201761802) derived	0.002				0.008	
Q159K (rs79402270) derived	0.016		0.04	0.003	0.05	0.002
**R173Q (rs747698893) derived**					0.0001	0.001
T184I (rs149938637) derived				0.001		
G207D (rs200520896) derived				0.001		
S225N (rs373051924) derived			0.001			
M237V (rs201413886) derived					0.001	
S242P (rs147552663) derived		0.002				
S246R (rs190314299) derived			0.001			
P263A (rs571820459) derived				0.001		
R272H (rs145237146) derived	0.001		0.004	0.003		
L282I (rs141979823) derived	0.003		0.004		0.01	
M288T (rs565811736) derived		0.001				
E329D (rs554234835) derived				0.001		
V333I (rs147275271) derived	0.002	0.005				0.001
E362Q (rs558823933) derived				0.001		
E362A (rs576618731) derived				0.001		
D369N (rs540873212) derived					0.001	
S384N (rs148498977) derived		0.001				
S389L (rs572239643) derived		0.001				
E402K (rs185655763) derived				0.001		
P419S (rs140275311) derived				0.001		
P419R (rs145278609) derived		0.001				
S427T (rs532733390) derived					0.001	
K463R (rs34661425) derived	0.02	0.06	0.01	0.002		
T469M (rs199734764) derived					0.001	
D478A (rs148156611) derived	0.001		0.003	0.001		
**N483T (rs574215585) derived**	0.004	0.001				0.02
N498S (rs373096835) derived	0.001	0.002				

I501T (rs146762807) derived	0.001	0.002				0.001

**ENAM** (over 494 missenses)	Allele frequency of the variant					
	**All**	**AFR**	**AMR**	**EUR**	**EAS**	**SAS**
I26F (rs141034810) derived	0.001				0.005	
M40V (rs533270579) derived		0.001				
Q88R (rs565258194) derived			0.001			
K111T (rs532584416) derived						0.001
K115N (rs544910871) derived						0.001
P125A (rs563240539) derived						0.002
P147L (rs201940627) derived			0.001			
P155L (rs374823061) derived		0.001				
Q171K (rs192104094) derived					0.002	
Q171R (rs556419641) derived				0.001		
Q178E (rs574769451) derived		0.001				
L180S (rs367993395) derived		0.002				
R187C (rs200947570) derived			0.001			
G195A (rs143129444) derived		0.001				0.001
G201V (rs574810587) derived						0.001
G239V (rs541993951) derived					0.002	
T251M (rs188769287) derived					0.001	0.001
R317C (rs564877872) derived						0.001
R332G (rs201174697) derived		0.001				
G358S (rs147295492) derived	0.001				0.003	
R368C (rs529994198) derived					0.001	
R373P (rs143134915) derived						0.001
P374L (rs566662520) derived				0.001		
A382V (rs144413018) derived					0.001	
**G389S (rs74511578) derived**	0.013	0.05	0.003			
Y394H (rs570864098) derived					0.002	
Y394F (rs142747446) derived	0.001			0.002		0.002
**T415I (rs779308123) derived**				< 0.0001		
**R429H (rs375758699) derived**	0.0005					
G442S (rs189446150) derived			0.003			
P450S (rs180899807) derived					0.002	
V466I (rs148712673) derived	0.002	0.001		0.007		
G478V (rs554084117) derived						0.001
V488L (rs572302644) derived		0.001				
S494Y (rs143617256) derived				0.001		
K518N (rs146719067) derived				0.001		
T533S (rs576885761) derived					0.002	
S542T (rs200201281) derived				0.002		
I552M (rs145361338) derived						0.001
P555L (rs201913549) derived					0.001	
F576L (rs2609428) derived	0.04	0.13	0.014	0.01		
P588S (rs149033201) derived		0.002				
G596R (rs201141678) derived						0.001
K633N (rs552101482) derived			0.001			
**T648I (rs7671281) derived**	0.82	0.47	0.89	0.95	0.98	0.94
V649L (rs199541879) derived				0.001		
E674K (rs139631708) derived					0.001	
H685R (rs535506506) derived			0.001			
S693L (rs147484294) derived			0.001			
P701S (rs539990971) derived			0.001			
P724L (rs3796703) derived	0.013	0.002	0.004	0.013	0.04	0.006
Y731C (rs181187768) derived	0.001					
R763Q (rs3796704) derived	0.14	0.37	0.1	0.05	0.02	0.05
Q775R (rs572270911) derived						0.001
R843S (rs74981230) derived	0.002				0.01	
D905G (rs199816355) derived				0.001		
T916A (rs552776312) derived						0.002
R946T (rs79087969) derived	0.007	0.03	0.001			
**T951M (rs561402393) derived**					0.0001	0.0001

R954K (rs552090369) derived				0.001		
N1008S (rs200932780) derived				0.001		
G1022V (rs556253149) derived						0.001
R1048I (rs535411629) derived			0.001			
D1076N (rs140345705) derived		0.001				
T1095S (rs576112196) derived		0.001				
Q1104Stop (rs114751362) derived		0.001				
S1107N (rs71599965) derived	0.003	0.002	0.003	0.01		0.001
I1108V (rs529369991) derived						0.001
F1121I (rs541159378) derived		0.001				
V1134L (rs200396980) derived	0.002					0.01
D1136H (rs142122738) derived	0.001	0.002	0.001			
**MGP** (over 55 missenses)	**Allele frequency of the variant**					
	**All**	**AFR**	**AMR**	**EUR**	**EAS**	**SAS**
**A8V (rs142330429) derived**	0.02			0.004	0.031	0.043
A12T (rs184536928) derived			0.001			
G30S (rs531889747) derived						0.001
H48N (rs550400563) derived					0.001	
N64H (rs145210821) ancestral	0.002	0.001	0.01	0.001		
A77S (rs536781673) derived		0.002				
K78E (rs1801716) derived	0.004		0.004	0.003		0.014
R84Q (rs536440407) derived						0.001
E92K (rs550754490) derived				0.001		
N94H (rs539042522) derived		0.001				
R119H (rs546998085) derived		0.001				
R125Q (rs145254250) derived	0.001	0.004				
**T127A (rs4236) ancestral**	0.39	0.55	0.4	0.38	0.13	0.43
