# Supplementary figures and images for: Neanderthal and Denisova tooth protein variants in present-day humans
Source: PLoS One. 2017 Sep 13;12(9):e0183802. doi: 10.1371/journal.pone.0183802 (PMC5597096; doi:10.1371/journal.pone.0183802)

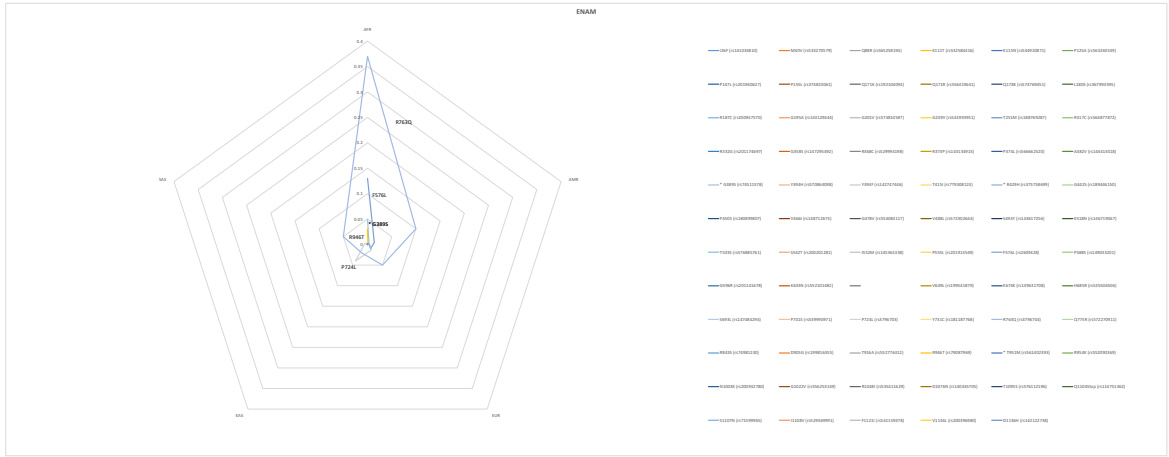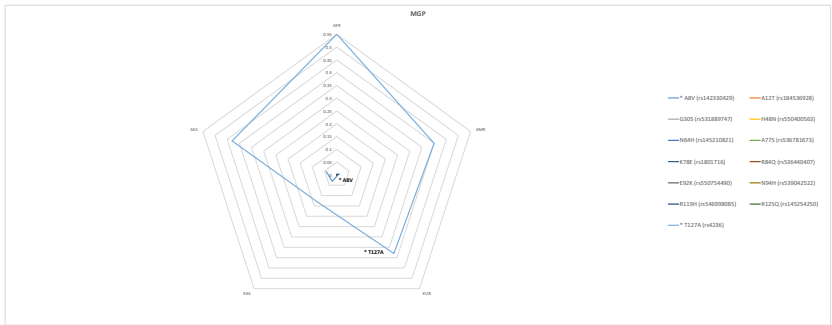

Supplement: S1 Fig — For clarity in the ENAM diagram, the major variant T648I was not considered. * Archaic variants. (PDF) [file pone.0183802.s002.pdf]

Frequency of archaic missense variants in living humans

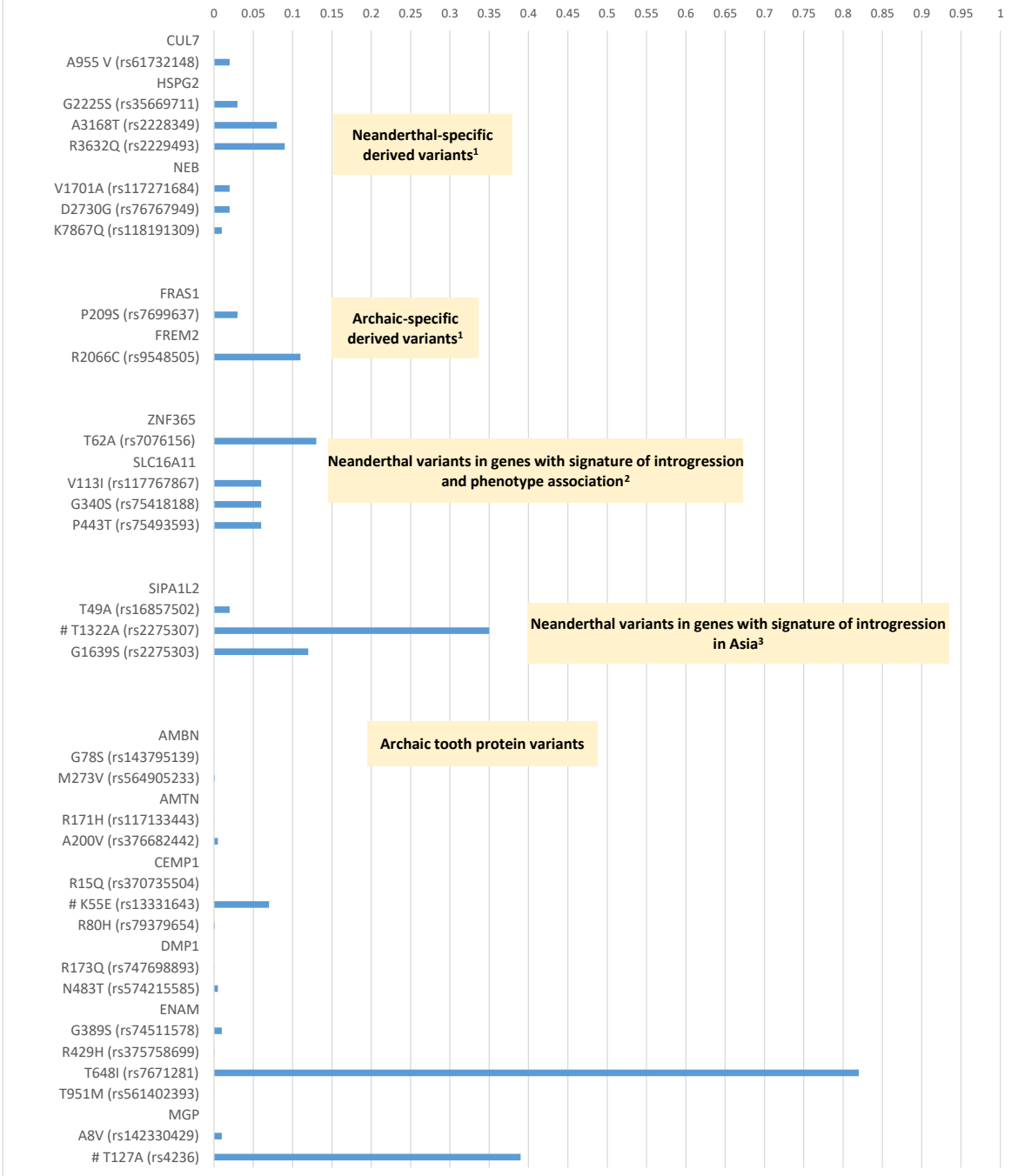

Supplement: S2 Fig — 1CUL7, HSPG2, NEB, FRAS1, FREM2 are found in Neanderthal or archaic (Neanderthal + Denisova) introgressed regions (Castellano et al. 2014). 2ZNF365 and SLC16A1 are found associated with Crohn's disease in Ashkenazi Jews and Type II diabetes in Latinos, respectively (Sankararaman et al., 2014). 3SIPA1L2 is present in haplotype with signature of adaptive introgression in Asia (Vernot and Akey, 2014). # Ancestral variants. (PDF) [file pone.0183802.s003.pdf]
